# The Consequences of DNA Damage in the Early Embryo Are Important for Practical Procedures in Assisted Reproduction

**DOI:** 10.3390/ijms262010031

**Published:** 2025-10-15

**Authors:** Vladimír Baran, Štefan Čikoš, Dušan Fabian

**Affiliations:** Institute of Animal Physiology, Centre of Biosciences, Slovak Academy of Sciences, Šoltésovej 4, 040 00 Košice, Slovakia; cikos@saske.sk (Š.Č.); fabian@saske.sk (D.F.)

**Keywords:** early embryo, DNA damage, prenatal diagnostics, assisted reproduction

## Abstract

The maintenance of gene integrity is important for all types of cells, but, in the case of early embryonic cells, it is absolutely essential. This is because it influences not only the further development of the embryo but also, in some respects, the offspring. The occurrence and incorrect repair of cellular abnormalities after DNA damage during this period are the primary causes of fetal developmental disorders. If DNA damage occurs in germ cells or the fertilized oocyte and the DNA lesions are not satisfactorily repaired, this can lead to the occurrence of chromosomal aberrations during early embryogenesis and eventually to genetic instability during embryonic development. This developmental ability is related to the level of the DNA damage. Therefore, examining the events related to DNA damage response at the sub-cellular levels is of the utmost importance. In this context, subcellular diagnostics of such events during the selection of embryos with the highest implantation potential applied in the practice of assisted human reproduction are key to successful outcomes. It is important to apply new relevant knowledge from basic research to clinical practice, as well as considering new technical possibilities or trends in this area. The aim of this review is to provide a general overview of the molecular events associated with DNA damage in the early embryo and to outline the possible use of this basic knowledge in assisted reproduction procedures.

## 1. Introduction

After long-term gametogenesis, which generates a haploid egg and sperm, a diploid zygote is created during the process of fertilization. This is the beginning of a dynamic period of cleavage embryo development, the course of which significantly influences the further development of the embryo. Early embryogenesis begins with fusion of highly differentiated gametes—oocyte and sperm. Unlike the oocyte, the sperm is fully developed at the time of fertilization. The development of the oocyte, which is arrested in metaphase II of the second meiotic division, continues after fertilization. Meiosis is completed with the extrusion of the second polar body. Immediately after fertilization, the zygote begins entering the first mitotic cycle. This process requires DNA modification at the histone level. Therefore, paternal DNA undergoes rapid active demethylation before S-phase. Maternal demethylation proceeds passively, coupled to DNA replication [1]. The result of this process is that the fertilized oocyte synchronizes its haploid set of chromosomes with the sperm, and, subsequently, male and female pronuclei are formed. The pronuclei differ not only in size but also in that the male pronucleus contains so-called protamines, which are replaced by full-fledged histones in the process of demethylation. These are contained in the cytoplasm of the oocyte, and this leads to the formation of a one-cell embryo [2,3]. The pronuclear bodies subsequently migrate to the center of the one-cell embryo and degrade its nuclear envelope. During migration, the first instance of DNA replication (S-phase) is initiated after fertilization. At the end of S-phase, one-cell embryos form two separate promitotic spindles around each set of parental chromosomes [4,5]. However, these spindles rapidly fuse to initiate the first cytokinesis after successful formation of the metaphase chromosomal metaphase configuration. Because one-cell embryos do not have centrosomes as defined in somatic cells, analogous structures called microtubule-organizing centers (MTOCs) are involved in spindle assembly in one-cell-stage embryos [6,7,8]. It has previously been suggested that this complicated formation of the first embryonic mitotic spindle results in a longer duration of the one-cell embryo stage [9], which may increase the susceptibility of one-cell embryos to errors in the mitosis process [10]. Subsequent cleavage of the embryo results in faster mitotic cycles; however, during the first few stages of the early preimplantation embryo, blastomeres divide without growth phases. Recently, blastomeres undergoing the main wave of embryonic genome activation were shown to have the highest levels of exon skipping compared with other tissue types. Moreover, exon skipping is mostly temporary and is restored shortly after the complete activation of the transition to embryonic genome expression. Thus, potential splicing failure certainly contributes to the attenuation of the DNA damage response during mammalian zygotic genome activation [11]. During these early cleavage stages, regulated translation utilizes mRNA transcripts of maternal origin but with gradual activation of the embryonic genome [12]. The embryo’s primary task is accurate genetic transmission to offspring. This presupposes, in the first phase, the successful resumption of mitotic activity after fertilization and the subsequent initiation of embryonic genome expression. Maintaining overall genomic stability is also important in the subsequent cleavage stages of the early preimplantation embryo because of the activation of the embryonic genome and the primary differentiation of totipotent blastomeres. In the context of the dynamics of epigenetic modifications during the proliferation of the early embryo [13], this embryo uses a different “strategy” to cope with DNA damage at early stages compared with somatic cells [14]. This is critical, as early embryonic instability carries disproportionately severe consequences. Compared with somatic cells, when these disorders are “solved” by eliminating such somatic cells within the tissue, such disorders in the embryo potentially have long-term consequences for embryogenesis or even the offspring [15]. In this sense, preimplantation embryos represent a very specific biological system because highly differentiated germ cells, oocyte and sperm, must remodel their chromatin structure and then successfully initiate the expression of the embryonic genome [16]. DNA damage at various levels also occurs under physiological conditions in connection with massive chromatin restructuring during the first embryonic mitosis. DNA damage in cycling cells can also be induced by so-called nonphysiological factors of endogenous or exogenous origin. If DNA damage occurs in germ cells (oocytes or sperm) or a fertilized oocyte and the DNA lesions are not satisfactorily repaired, chromosomal aberrations can occur during early embryogenesis and eventually lead to genetic instability during subsequent embryonic development [17]. DNA lesions occur more frequently in mature sperm than in mature oocytes [18]. Oocytes are capable of efficient DNA repair, which is important for ensuring fertility [19]. Unlike oocytes, sperm do not have the same ability because they lack the molecular content necessary for DNA repair. Although sperm DNA damage does not compromise fertilization, it increases DNA damage in the paternal pronucleus of the zygote. Therefore, the repair of sperm DNA damage appears to be a key factor in ensuring the correct development of early embryos [20].

## 2. First Mitosis and Cell Cycle Control

The course of early embryogenesis significantly influences not only the further development of the embryo but also, in some respects, the offspring. The resumption of the mitotic cycle in one-cell embryos and complete transition from maternal to embryonic transcriptional control ensure the further successful course of embryogenesis [21]. In this process, the replacement of sperm protamines with full-fledged histones of maternal origin is of paramount importance. However, both male and female pronuclei retain some parental-specific histone methylation patterns in pericentromeric heterochromatin regions. During the first stages of early embryogenesis, both parental genomes undergo epigenetic reprogramming via a demethylation process, which is dependent on DNA replication [22]. This process of paternal DNA demethylation typically creates DNA breaks, but fertilized oocytes have the potential to efficiently repair these lesions during the first cell cycle to prevent chromosome fragmentation, thereby eliminating potentially undesirable consequences [23]. mRNA transcripts of maternal origin are required for the control of resumption and the course of the mitotic cycle and the transition from maternal to embryonic control of gene expression. The timing of the main wave of embryonic genome expression varies between species but occurs completely at the preimplantation embryo stage. For example, in mice, minor activation occurs at the middle to late S-phase of the one-cell stage, and major activation occurs at the four- to eight-cell stage. In other species, it occurs later, such as at the eight- to sixteen-cell stage in cows [24]. The uncoupling of morphogenetic and transcriptomic events is also species specific. For example, while morula compaction occurs at the eight-cell stage in both human and mouse embryos, the main wave of embryonic genome activation occurs at the eight-cell stage in humans but at the two-cell stage in mice. Subsequent mitotic divisions after the first longer mitotic cycle occur rapidly. Here, the cell cycle oscillates between the phases of DNA synthesis and mitosis with an incomplete checkpoint mechanism [25]. In the later phases, during gastrulation, mitotic cycles slow down with the inclusion of the so-called gap phase and cell cycle checkpoints [26,27]. The variability of the dynamics of the cleavage of individual blastomeres may affect the coordination mechanism to a greater extent in the cleaving embryo [28]. The fast mitotic cycles of blastomeres in the early stages of embryogenesis are synchronous compared with the later developmental phases of the preimplantation embryo when these dynamics slow. With slowdown, the cell cycles of blastomeres begin to desynchronize even before gastrulation [29]. The extension of the cell cycle is apparently related to the synthesis of embryonic-derived proteins after the elimination of all maternal supplies [30]. The correct completion of this process is crucial for the primary differentiation of totipotent blastomeres into embryonic and trophoblast stem cells before implantation into the uterus. In addition, it significantly affects the development of the embryo and becomes an important criterion in the evaluation of the quality of assisted reproduction technology [31]. Preimplantation embryo present greater levels of chromosomal abnormalities during early cleavage stages compared with later stages [32]. Thus, preimplantation embryos can already acquire an aneuploidy phenotype in early developmental stages, which points to the fact that these first mitotic cycles are more susceptible to chromosomal aberrations [33]. Monitoring events during the cell cycle related to gene expression is therefore a fundamental aspect of the cell cycle. In this process, every embryonal cell has a control mechanism called a cell cycle checkpoint [34,35]. Cell cycle checkpoints play a key role in regulating the cell cycle during early embryonic development and influence the integrity of the genome and events that have a fundamental impact on the further development of both preimplantation and post-implantation embryos. Monitoring the cell cycle is particularly important in one-cell embryos, as this cycle initiates the first mitotic cycle after fertilization. This resumption of mitosis is conditioned by a massive substantial reorganization of both male and female chromatin. The cell cycle control mechanism involves three main checkpoints defined as G1/S, intra-S-phase, G2/M and the spindle assembly checkpoint (SAC) that are involved in controlling the course of mitosis in specific substages. The G1/S checkpoint is very sensitive to damaged DNA and therefore inhibits S-phase entry by affecting the activity of cyclin-dependent protein kinases. The so-called intra-S checkpoint is also interpreted in connection with the initiation and course of the S-phase. This directly regulates replication elongation to maintain the integrity of stalled replisomes [36]. Recent observations document the absence of a replication timing program in the first two cleavage cycles of the mouse embryo. The entire genome replicates gradually and slowly in one- and two-cell embryos. Chromosome segregation errors are more common at these embryonic stages, whereas the acceleration of replication forks during the four- and eight-cell stages ensures sufficient nucleosides [37]. Thus, in the early cleavage stages, the embryo undergoes a transient period of genomic instability, which is related to the replisome level and regulation of the timing of DNA replication. This phenomenon likely affects the overall stability of the genome in the early stages of preimplantation embryo development. However, accurate genome replication during S-phase is of fundamental importance, especially in single-cell embryos. Double-strand DNA breaks are the most serious type of DNA damage because they induce chromosomal instability and fail at chromatin remodeling [38]; therefore, the intra-S-phase checkpoint is highly important. Patterns of DNA replication, as the basis of genome stability are established in mammals very early in embryogenesis and are related to patterns of nuclear organization in germ cell lineage of the embryo. Therefore, studies of DNA replication and genome stability in early mammalian embryos are important for understanding both normal and deviant genetic variation and for elucidating the fundamental principles of genome regulation [39]. The G2/M checkpoint represents the final cell cycle checkpoint before entry into mitosis. Its activation arrests the cell in the G2 stage if some chromosomal aberrations are detected. The SAC represents the main checkpoint involved in the onset of cytokinesis. It is activated during the metaphase–anaphase transition and prevents premature separation of sister chromatids, thus delaying the onset of anaphase [40]. Cell-cycle checkpoints chiefly safeguard DNA integrity for faithful genetic transmission. The response to DNA damage is reversible inhibition of the cell cycle to allow DNA repair. After a rapid response to DNA damage and subsequent successful repair, the checkpoint is turned off, and the cell cycle resumes [41]. If DNA damage is not successfully repaired, it can have serious consequences not only for the further course of the cell cycle of embryonic cells but also for the development of the embryo as a whole [42]. The physiologically given frequency of the cell cycle in the cleaving embryo positively correlates with its viability. However, in the case of stress or adverse conditions, the embryo can stop or slow its development and induce a state of “protective diapause”.

## 3. DNA Damage and Response

**Figure 1:**
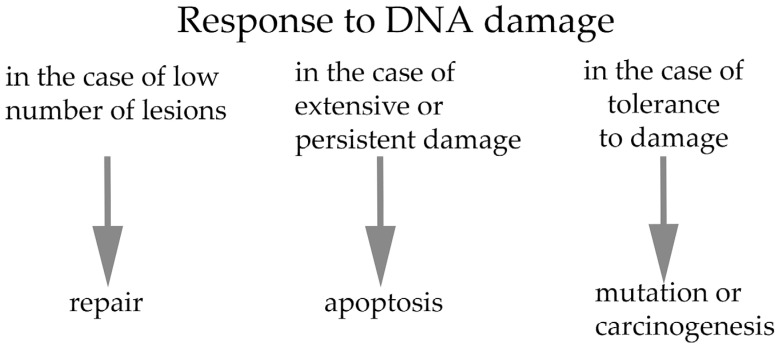
The response to DNA damage can, in principle, lead to three possible outcomes: (i) repair of DNA damage; (ii) cell death mediated by activation of the apoptotic pathway; or (iii) mutation or eventual carcinogenesis.

Zygotes possess the potential to rapidly recognize damaged DNA structures and induce a rapid response in these lesions. This provides activation of the antiapoptotic machinery and an opportunity for DNA repair, with subsequent continuation of the first mitotic phase [43]. The resumption of mitotic activity after fertilization of the oocyte occurs in the inherent maternal environment until the initiation of embryonic genome expression. Nevertheless, cell cycle control checkpoints are limited in fully grown oocytes, which allows oocytes with DNA damage to resume meiosis unless the damage levels are severe [44,45]. However, early embryos have the potential to successfully repair such damage during the first cell cycle after fertilization to prevent chromosome fragmentation, embryo loss and infertility [23]. Despite a certain degree of tolerance of maturing oocytes and very early embryos to DNA damage, cell cycle signaling pathways are crucial for the activation of downstream effectors that control the integrity of embryonic genomes. In this context, early embryos overcome DNA damage using a different “strategy” than that utilized by somatic cells [14]. It was demonstrated that oocytes exhibiting moderate levels of DNA damage complete maturation despite a delay in chromosome rearrangement at anaphase I. These results confirmed that DNA damage in maturing oocytes does not activate the G2/M checkpoint but allows meiosis progression. It has been shown that mild DNA damage before entry into the first S-phase is tolerated, suggesting that completion of the first cleavage stage takes priority over potential elimination of the fertilized oocyte, even with an unreplicated portion of DNA [46]. The response to DNA damage can, in principle, lead to three possible outcomes: (i) repair of DNA damage; (ii) cell death mediated by activation of the apoptotic pathway; or (iii) tolerance to the lesion, which can lead to mutation or eventual carcinogenesis [38]. Because several overexpressed embryonic genes are involved in DNA repair, the repair of damaged DNA appears to be the primary response of the early embryo [47]. In the case of extensive or persistent DNA damage, embryonic death is the last resort to “protect” genomic integrity [48]. After successful DNA repair, the checkpoint is turned off, and the cell cycle resumes [41]. In one-cell embryos, when the mitotic cell cycle resumes, accurate genome replication during S-phase is essential. In this context, double-stranded DNA damage is considered the most serious type of DNA damage, because it can cause chromosomal instability [38]. In this case, incorrect replication often leads to serious consequences, such as implantation failure, spontaneous abortion, genetic disease, or embryonic death [42]. The development of the human embryo is more sensitive to the consequences of DNA damage than is the case in early mouse embryos [49,50,51]. This is likely related to evolutionary differences, with mouse embryos being more efficient at protecting their DNA integrity [52]. The continuation of development of one-cell-stage embryos with some degree of DNA damage, allows for a certain degree of tolerance of cell cycle checkpoints. This property of the early mammalian embryo allows it to complete the entire cleavage process until the formation of fully functional apoptotic machinery in the blastocyst before implantation [53]. The blastocyst is an important stage in the preimplantation development of the embryo because, after primary previous differentiation into supporting and embryonic stem cells, it has all the mechanisms necessary to recognize and eliminate damaged blastomeres [54]. However, such DNA lesions followed by proper repair may promote genomic diversity in response to endogenous/exogenous causes. A high degree of genomic instability caused by a low level of repair of damaged DNA can lead to congenital disorders caused by chromosomal abnormalities [55]. In this context, approximately half of the blastocysts contain genomic alterations that cause a significantly high incidence of pregnancy loss [56]. Recent findings establish DNA damage tolerance thresholds as crucial safeguards of genome integrity during germline development [57]. Early embryos have the ability to repair damaged sperm DNA by activating the cellular repair machinery during the first mitotic cycle. However, the effectiveness of this mechanism is limited by female age, as maternal mRNA levels decline with increasing maternal age. This leads to reduced efficiency of DNA repair and negative downstream effects on embryonic development [58,59,60,61]. However, efficient repair of damaged DNA molecules in gametes or even in early embryos is crucial for the subsequent genomic stability of the embryo. In this context, it is important to further understand the mechanisms of these molecular processes with respect to the biological specificity of sperm/oocytes and preimplantation embryos. A more thorough examination of these mechanisms will ultimately aid in the development and management of fertility in older women [62]. It was documented that sperm DNA damage can still fertilize oocytes with negative effect on early embryonic development following in vitro fertilization [63]. In this case, the embryo is able to use a range of mechanisms to repair DNA damage; however, this ability is limited. Unique checkpoint activation and cleavage delay are required to allow for a lengthy process, i.e., DNA repair because DNA repair genes and proteins are temporally expressed. The result is aneuploid mosaicism of the early embryo, leading to termination of embryogenesis or congenital defects when such embryos escape developmental arrest [64,65]. In previous study, we demonstrated that oocytes containing a mean degree of DNA damage are able to complete maturation despite an increased number of lagging chromosomes emerging in anaphase I, resulting in chromosomal fragment cell phenotypes at the end of oocyte maturation (metaphase II) [46]. However, it has been shown that oocytes with high levels of DNA damage fail to successfully complete the process of maturation during meiosis [44]. These results confirm that DNA damage in fully grown oocytes does not activate the G2/M checkpoint and allows progression in meiosis I. Finally, there is limited knowledge regarding the activity and “effectiveness” of cell cycle checkpoints in oocytes as well as early embryos. It was assumed that tolerance to DNA damage must be low during this critical stage of development [38]. Possible cellular responses to DNA damage are summarized in Figure 1.

## 4. Consequences on Early Embryogenesis

**Figure 2:**
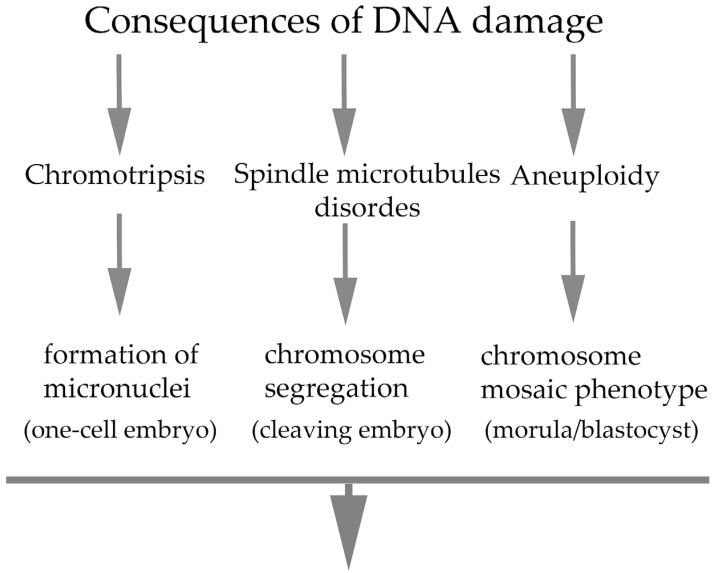
Simplified scheme of the consequences of DNA damage in embryos during early cleaving stages and implantation period. The developmental stages at which the phenotypes have been most frequently detected and indicated in parentheses (corresponding representative references: [46,73,74,75,78,79,86,87]).

All the cellular abnormalities that occur during the early cleavage stages are the primary cause of defects in subsequent fetal development. DNA damage is considered a serious cause because it often results in failure of embryonic development. In the context of the response to DNA damage at the cellular level, phenotypic changes in embryonal cells have been well documented. Several studies have shown that preimplantation embryos respond to damaged DNA in sperm or oocytes or to DNA damage during the early stages of cleavage but only with a certain degree of tolerance [34,66,67,68,69,70]. The zygote tolerates a certain degree of DNA damage and considers it a priority to complete the first cleavage stage and continue embryogenesis as long as possible. This tolerance phenomenon has been defined as a “checkpoint adaptation” in human cancer cells in the context of pharmacological treatment. Despite the adaptive nature of preimplantation embryos, statistics in multiple studies document a high rate of infertility in the field of human reproduction [71,72]. In connection with this adaptation, the formation of micronuclei of various sizes [73,74] associated with the process of chromothripsis was observed, which often leads to genomic instability [75] as a potential condition for the occurrence of cellular mutation [76]. Multinucleation was the most frequent abnormal mitotic event during embryo development. This process depends on the stage of embryogenesis when multinucleation occurs. Compared with four-cell multinucleated embryos, multinucleated two-cell embryos are more common in blastocyst development [77]. However, experiments have revealed a direct association between chromosome segregation defects and micronucleus formation [78,79]. In addition, three micronucleus phenotypes have been defined on the basis of their subcellular topology [80,81], but the process or conditions for the possible integration of micronuclei into complete daughter nuclei have not been studied to date. Although the cellular impact of micronuclei formation is less understood, we propose that the occurrence of micronuclei in early embryos is related to DNA replication disorders following the incorrect attachment of spindle microtubules to kinetochores during the first embryonal mitosis [46]. The cleavage kinetics of early embryos may not be affected; in the case of chromosomal segregation associated with the formation of micronuclei in later stages of preimplantation embryo development, there is a greater probability of embryogenesis failure with subsequent abortion [82,83]. In addition, in accordance with observations in somatic cells, Vázquez-Diez et al. [84] reported that micronuclei can persist in cells for several cleavage stages during embryonic development without contacting the blastomere nucleus. This may contribute to the high frequency of aneuploid cells in mammalian embryos but may also serve to insulate the embryonic genome from the effects of chromothripsis. Interestingly, the micronuclei in DNA damage-positive embryonic cells lacked DNA replication activity, in contrast to those in human somatic cells, in which only weak DNA replication in micronuclei has been documented. This replication activity is inefficient with respect to the course of mitosis and is also asynchronous with that of the nucleus [85]. DNA damage in the early stages of embryogenesis often manifests as the development of the so-called chromosomal mosaic phenotype in the morula or blastocyst, which is caused by the presence of aneuploid blastomeres [86,87]. This is due to a high rate of aneuploidy of mitotic origin and resulting chromosome segregation disorders. It has been shown that 20 to 30% of such blastocysts exhibit a mosaic phenotype [64,88,89]. However, early cleavage of damaged DNA does not necessarily cause the failure of embryogenesis beyond the blastocyst stage. However, the selection of embryos that have reached the blastocyst stage is key in choosing embryos with the most potential for further development [82]. In this context, the status of embryos immediately before implantation is very important. During the cleavage stages of the preimplantation embryo, newly synthesized repair proteins generate fully functional apoptotic machinery that can efficiently remove damaged blastomeres [90]. This finding is consistent with our data on reduced blastomere numbers in blastocysts because of prior DNA damage [46]. Complete arrest of embryonic development has been documented at critically supra-threshold levels of DNA damage [91]. The threshold tolerance of early cleavage embryos also refers to a lower level of ribonucleotide incorporation into DNA, although this can potentially lead to developmental defects [92] and possible depletion of some differentiated blastomeres, especially from the embryonic stem cell region of the so-called inner cell mass [93]. The process of apoptosis can mediate the elimination of problematic blastomeres to achieve nearly embryo undergoing physiologically stable proliferative. Consequences of DNA damage in embryos are summarized in Figure 2.

### Assisted Reproduction

Despite the significant progress achieved in the field of assisted reproduction, more than 50% of embryos produced in vitro still do not reach the stage of a biologically full-fledged blastocyst [94]. According to published estimates, approximately half of human blastocysts exhibit alterations in the embryonic genome, which are the cause of a high incidence of pregnancy loss [95]. However, the presence of blastomeres with fragmented chromatin may not be an indicator of unacceptable blastocyst quality, as early embryos at this stage of development may successfully eliminate such abnormal cells to preserve their developmental potential [80]. However, blastocysts with acceptable morphology may not be able to be implanted successfully and produce healthy offspring [95]. Experiments have revealed that a self-regulatory mechanism in the sense of eliminating problematic blastomeres exists [96]. It is important to clarify the principles of these mechanisms to optimize procedures in the practice of assisted reproduction. It has been statistically documented that embryos classified as mosaic embryos have greater potential for miscarriage compared with euploid embryos, shortly after implantation. However, children born from mosaic embryo transfers are similar to children born from euploid embryos. Prenatal testing in this setting has suggested that mosaicism will resolve in the majority of pregnancies, although this process may not be completely effective. In a small percentage of cases, mosaicism has persisted throughout pregnancy [97]. These findings provide a rationale for weighing the risks versus benefits of embryo transfer in preimplantation embryos with a mosaic phenotype. Selection of embryos should meet several criteria. In embryo selection, mainly morphological criteria were defined. However, the use of morphokinetic embryonic characteristics using time-lapse systems and artificial intelligence in the application of invasive testing for aneuploidy (PGT-A) and noninvasive methods (niPGT-A) is important [98]. Despite these advances in embryo selection methods, the overall success rate of in vitro fertilization techniques remains between 25% and 30% [99]. The accurate diagnosis of so-called blastocyst mosaicism as a result of mitotic abnormalities is quite problematic [100] because of the insufficient understanding of the complexity of embryo genetics. This is related to the application of PGT-A testing, which is based on biologically uncertain assumptions and unvalidated guidelines, leading to the possibility of disposing of embryos with pregnancy potential [101]. It appears that the cytogenetic constitution of whole embryos may provide a more accurate picture of the physiological state of the blastocyst. The low rate of mosaic aneuploidy in high-quality blastocysts supports mosaic embryo transfer in patients without euploid embryos [102]. It was shown that up to 50% of human embryonic stem cells can survive due to increased expression of regulatory and repair factors [103]. This may provide the necessary amount of information for further optimization of the existing standardized process of in vitro methods and improvement of the rate of successful clinical pregnancies in the medical field of assisted reproductive technology.

The integration of damaged sperm DNA into the embryonic genome can lead to replication, transcription and translation errors in the cleavage embryo. The possible developmental consequence of this damage may manifest itself in the early stages of embryonic development [104] or later as delayed embryo development, which may impair the timing of implantation [105]. However, the primary source of aneuploidy is considered to be the occurrence of incorrect chromosome segregation in the oocyte during meiosis. The consequences of advanced and uncompensated aneuploidy significantly affect implantation and may ultimately be the cause of spontaneous abortion or birth defects [106]. Aneuploidy is a major obstacle to achieving successful pregnancy in assisted reproduction practice. In reproductive-aged women, the rate of aneuploidy in oocytes reaches 20–30%. In human sperm, it is only 1–8%. While the effects of maternal age on embryo chromosomal aneuploidy are well established, the impacts of male age and sperm quality on ploidy are less well defined [107]. Approximately 50% of oocytes from women of advanced age (40 years and older) areaneuploid oocytes [108]. Given the origin of aneuploidy as a phenotypic marker, two types of aneuploidy can be distinguished: (1) meiotic aneuploidy and (2) mitotic aneuploidy. Meiotic errors usually result in uniformly aneuploid embryos, whereas mitotic errors often cause mosaic morula phenotypes [88] that may persist into the blastocast stage [109]. The frequency of aneuploidy in oocytes and early embryos of mammals is high compared with that in somatic cells and increases with maternal age [110,111,112,113,114,115,116] Although the in vitro conditions of early embryo culture for IVF significantly increase the frequency of aneuploidy [117], even in the natural environment in vivo, these disorders are not much less common than would be expected [118]. In vivo mouse embryos presented aneuploidy rates per blastomere ranging from 4% in the zygote to 7% in 8-cell embryos, with a steep increase to 11% between 8- and 16-cell embryos [119,120]. Similar frequencies have been reported in embryos of other mammalian species, including humans [121], cattle [117,122], pigs [123] and rhesus monkeys [79]. In the later stages of the preimplantation embryo, when the embryo has overcome a manageable level of DNA damage, the presence of several aneuploid blastomeres determines the development of the so-called mosaic phenotype of the embryo. It has been shown that aneuploid blastomeres in the embryo survive and are able to divide; they are not eliminated often until hatching [93,118]. However, the implantation and subsequent differentiation of embryonic stem cells and the development of the embryo itself may be compromised, although a lower or acceptable number of aneuploid blastomeres in the embryo provides the possibility for subsequent successful embryogenesis [124,125]. However, insufficient mitotic “timer mechanisms” can significantly complicate the course of early embryogenesis because aneuploid blastomeres tend to prolong their mitotic cycle, which increases the likelihood of incorrect chromosome segregation due to weakened cohesion [126]. This phenomenon is probably related to the activation of the SAC checkpoint in an environment where the embryonic genome is already fully activated, i.e., the effect of the cell cycle control system is in full “condition” compared with the first cleavage cycles. The situation may be complicated by chromosomal fragments, i.e., micronuclei located outside the mitotic spindle and resulting from previous DNA damage [46]. The chromosomal fragments are covered by a membrane-like envelope [79,80,127,128] and they do not replicate “their” DNA and contain damaged DNA [46]. Unlike those in somatic cells, micronuclei in embryos do not contribute to chromothripsis [85].

**Figure 3:**
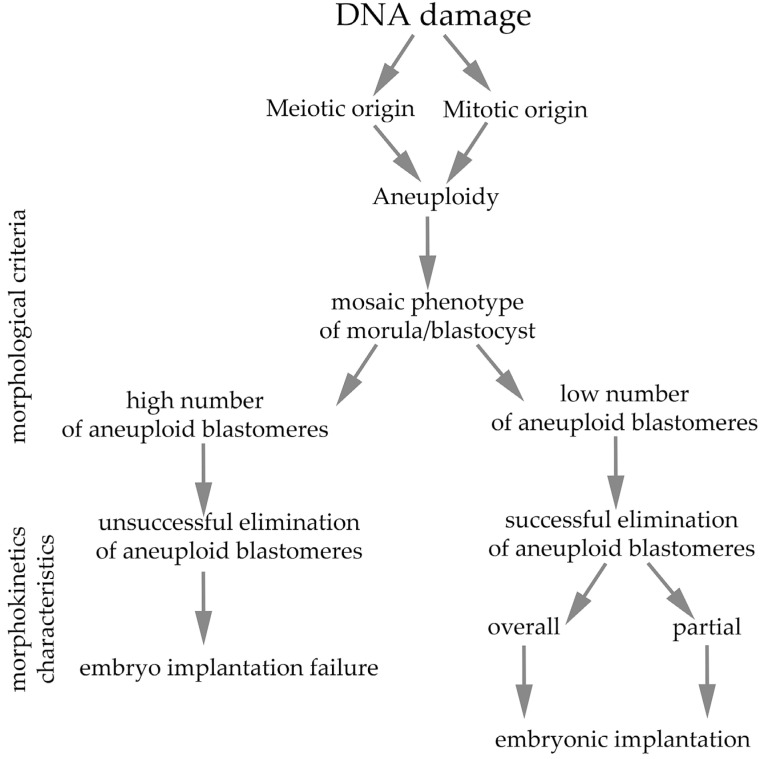
Phenotypes of blastocysts induced by DNA damage during meiosis or first mitosis and subsequent implantation outcomes. The relevant diagnostic approaches are indicated.

As already documented, meiotic aneuploidy is significantly influenced by the mother’s age [116,129,130,131], where dysfunction of mitochondrial processes also likely contributes to reproductive aging [132], unlike in males, where age does not play such a significant role [133,134]. In connection with these findings, mitochondrial supplementation is considered a potential therapeutic tool to slow oocyte aging [135,136,137,138]. Although there are various causes of aneuploidy, this phenomenon is highly complex and often leads to developmental arrest, implantation failure, or spontaneous abortion in both natural and assisted reproduction [139]. Phenotypes of blastocysts induced by DNA damage and subsequent implantation outcomes are summarized in Figure 3.

## 5. Conclusions

In assisted reproduction, embryos prepared for embryo transfer are evaluated on the basis of the dynamics of their development and basic morphological characteristics. However, the viability or biological quality of in vitro cultured embryos may not correlate with their microscopic appearance. An important unresolved question is how the occurrence of aneuploidy correlates with changes in the overall morphology of early embryos at the cellular level. Time-lapse imaging technology offers more precise quantification of cell kinetics compared with assessment of static preimplantation status. This technology confirms that the rate of embryo development and morphological parameters reflect the cytogenetic state of the embryo. Ultimately, a detailed understanding of the molecular mechanisms of DNA damage repair in both sperm and oocytes will contribute to the development of sophisticated therapeutic methods to improve fertility in individuals who are potentially healthy but also of advanced reproductive age [62].

## Data Availability

No new data were created or analyzed in this study. Data sharing is not applicable to this article.
